# GenomicusPlants: A Web Resource to Study Genome Evolution in Flowering Plants

**DOI:** 10.1093/pcp/pcu177

**Published:** 2014-11-27

**Authors:** Alexandra Louis, Florent Murat, Jérôme Salse, Hugues Roest Crollius

**Affiliations:** ^1^Ecole Normale Supérieure, Institut de Biologie de l’ENS, IBENS, Paris, F-75005 France; ^2^CNRS, UMR 8197, Paris, F-75005 France; ^3^Inserm, U1024, Paris, F-75005 France; ^4^INRA/UBP UMR 1095 GDEC (Génétique, Diversité et Ecophysiologie des Céréales), Clermont Ferrand, France

**Keywords:** Ancestral reconstruction, Evolution, Flowering plants, Genomics, Synteny

## Abstract

Comparative genomics combined with phylogenetic reconstructions are powerful approaches to study the evolution of genes and genomes. However, the current rapid expansion of the volume of genomic information makes it increasingly difficult to interrogate, integrate and synthesize comparative genome data while taking into account the maximum breadth of information available. GenomicusPlants (http://www.genomicus.biologie.ens.fr/genomicus-plants) is an extension of the Genomicus webserver that addresses this issue by allowing users to explore flowering plant genomes in an intuitive way, across the broadest evolutionary scales. Extant genomes of 26 flowering plants can be analyzed, as well as 23 ancestral reconstructed genomes. Ancestral gene order provides a long-term chronological view of gene order evolution, greatly facilitating comparative genomics and evolutionary studies. Four main interfaces (‘views’) are available where: (i) PhyloView combines phylogenetic trees with comparisons of genomic loci across any number of genomes; (ii) AlignView projects loci of interest against all other genomes to visualize its topological conservation; (iii) MatrixView compares two genomes in a classical dotplot representation; and (iv) Karyoview visualizes chromosome karyotypes ‘painted’ with colours of another genome of interest. All four views are interconnected and benefit from many customizable features.

## Introduction

Comparative genomics combined with phylogenetic reconstructions is becoming increasingly important as more genomes are being sequenced. Moreover, our current understanding of biological processes is limited to contemporary ones occurring in living organisms. Yet biology is a historical science: all current biological processes are the result of complex evolutionary events. Large-scale genome sequencing makes it possible, through comparative genomics, to gain knowledge on the ancestral biological genome organization that preceded and laid the ground for today’s biology.

Efficient visualization tools are needed to explore and interpret these genomic data. Comparative genomics is a complex field because of the different dimensions researchers have to explore, such as spatial organization (related to gene position), or temporal relationship (related to gene and genome evolution). Bioinformatics tools are available to visualize and compare genomes (López and Samuelsson 2011, Soderlund et al. 2011). Some are dedicated to plant genomes (Brendel et al. 2007, Courcelle et al. 2008, Duvick et al. 2008, Rouard et al. 2011, Goodstein et al. 2012, Van Bel et al. 2012, Guo et al. 2013), but most are restricted to two or three genomes at a time and few provide access to ancestral gene organization (Byrne and Wolfe 2005, Muffato et al. 2010).

Since the sequencing of the two first models of plants organisms (Arabidopsis Genome Initiative 2000, International Rice Genome Sequencing Project 2005), angiosperms became new models organisms to elucidate the mechanisms and the consequences of polyploidization in eukaryote evolution (Lyons and Freeling 2008, Renny-Byfield and Wendel 2014).

Here we present a release of the GenomicusPlants server, previously described with its counterpart dedicated to the Vertebrate, Fungi, Metazoan and Protists clades (Louis et al. 2013). This update focuses on flowering plants, with the reconstruction of ancestral gene content and order, and the availability of new display tools.

## Database Construction and Content

### Data sources

**Fig. 1:**
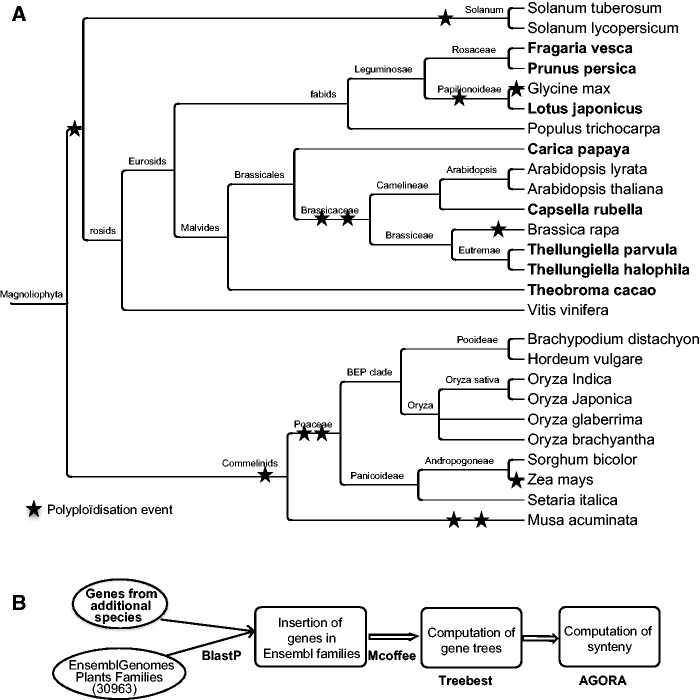
(A) A cladogram of the 26 extant angiosperms available in GenomicusPlants, and the 23 reconstructed ancestors. Species added in the families downloaded from EnsemblGenomes are in bold. Branches do not represent time scale. Branch labels indicate the ancestor of each clade. Stars represent the different polyploïdization events [according to the PGDD database (Guo et al. 2013)]. (B) Schematics of the bioinformatics pipeline used to integrate the additional extant species in the EnsemblCompara GeneTrees and to reconstruct phylogenetic trees.

As a starting point for comparative genomics and ancestral gene order reconstruction, we selected 18 species from the EnsemblGenome database release 16 (Kersey et al. 2014), comprising 10 monocotyledons and eight dicotyledons. For these extant species, all protein-coding sequences were downloaded, together with their gene location and the gene family they belong to. Within these pre-existing families, we then integrated the data from eight other dicotyledons, downloaded from Phytozome8 (Goodstein et al. 2012) or from specific servers (see Table 1). This leads to a total of 26 extant genome species, and their 23 inferred ancestors (see Fig. 1).

**Table 1:** Additional species protein-coding gene content and comparison with other monocotyledons or dicotyledons from the EnsemblGenome database

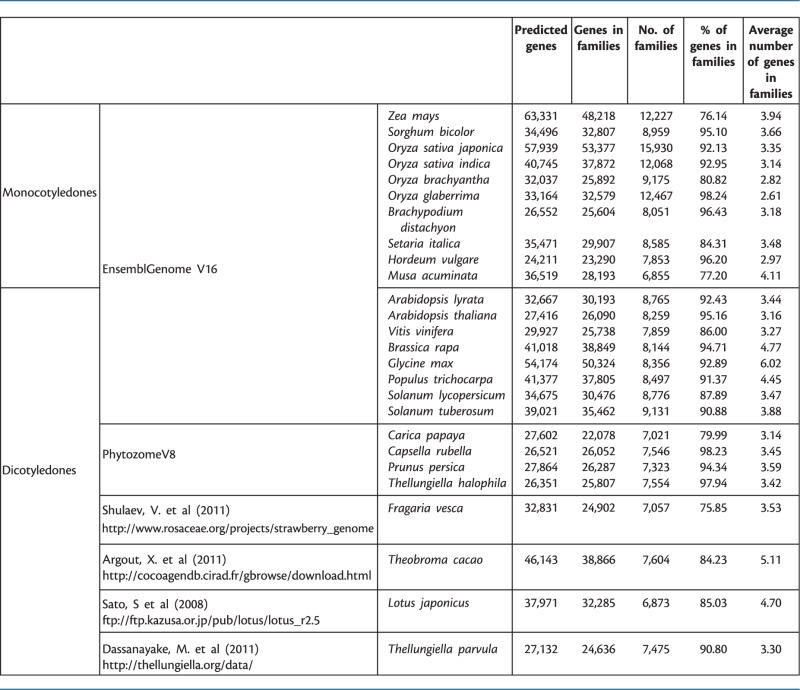

### Gene family computation

We used the Ensembl API to extract 30,963 sub-gene trees containing proteins of some or all of the 18 angiosperm genomes listed in Table 1. Each tree constitutes a family of orthologous and paralogous plant genes.

To integrate genes from additional species into these families were carried out the following steps. (i) We performed an all-against-all BLASTP comparison against predicted protein sequences from each species. BlastP results were filtered with a cut-off *P*-value of 10e-04. (ii) We calculated the average family bitscores between a given family of protein sequences and the protein of the additional species of insert. If a protein is associated with several families, we chose the family with the highest average bitscore. The results of this computation are given in Table 1. (iii) We built new multiple sequence alignments using M-Coffee (Wallace et al. 2006) and built a new phylogenetic gene tree reconciled with the species tree using the Treebest pipeline (Vilella et al. 2009).

### Ancestral genome inference

Ancestral genome are inferred by a method called AGORA (Algorithm for Gene Order Reconstruction in Ancestor) (Muffato 2010). The first step of this algorithm is to infer the gene content of each ancestor based on the phylogenetic trees computed by TreeBest and reconciled with the species tree. Once each ancestral gene content is inferred, parsimonious scenarios are deduced on pairwise comparisons of gene order between all pairwise genomes that are informative for the ancestor of interest (the ancestor is on the path between both species in the tree). Two modern genomes share a common ancestor, from which they both independently inherit some degree of conserved gene organization. Locally, this conserved gene organization can take the form of conserved gene adjacencies, where two genes a1 and b1 in species 1 are adjacent, and their respective orthologs a2 and b2 in species 2 are also adjacent. This definition of conserved gene adjacency may be more or less constrained. For example, it may request that the two adjacent genes also conserved their transcriptional orientation, or it may tolerate that some intervening genes separate the two genes as long as they did not exist in the common ancestor (i.e. lineage-specific genes). AGORA first filters the two genomes to retain only genes present in their last common ancestor. It then intersects the two genomes to retain the pairs of adjacent genes in the same transcriptional orientation, which will be considered as potentially ancestral. Finally, it assigns each potential ancestral pair not only to the last common ancestor of the two species, but also to each ancestor along the branches that connect the two genomes, as long as no lineage-specific gene has been inserted between the two genes considered. Gene adjacencies inferred should all be ancestral if no events have taken place in any lineage. In this case, all genes should be involved in at most two adjacencies (one upstream and one downstream of its own position in the ancestral genome). Rearrangements, and gene gains and losses, however, create situations where a given ancestral gene is involved in additional adjacencies, thus creating a need to decide which adjacency is the most likely to be ancestral. To do this, the method labels each adjacency with a weight reflecting the number of times that it has been reported as conserved since this ancestor, through relevant pairwise comparisons of extant genomes according to the species phylogeny. Adjacencies are ranked by decreasing weight, and selected in turn from most to least conserved. By selecting these adjacencies, gene pairs are sequentially classified as ancestral as long as they are not already involved in a conserved adjacency with a higher weight. For a given ancestor, all ancestral genes that are identified as conserved neighbors in at least one such comparison become linked nodes in a graph. A weight reflecting the number of times this situation was observed in all the comparisons is then applied to each link.

At this stage, inconsistencies may appear in the form of ancestral genes connected to more than two neighbors. To resolve these, the weighted graph is processed using a top-down greedy algorithm where the links of highest weight are selected first and are used to select the most likely gene–gene connection in th ecase of multiple choices.

This produces a set of linear paths in the graph connecting ancestral genes based on the number of times their respective descendants are observed as extant neighbors. After extraction of the linear paths for each ancestor (that we call contigs), a second round of AGORA is made, by considering contigs as units of comparison. The algorithm then compares contig adjacencies in each pairwise extant genome that is informative for an ancestor, and builds a graph of contig adjacencies. The graph is then linearized to obtain final blocks (or scaffold) of ancestral gene order.

Reconstructed ancestral genomes vary in completeness (see ‘statistics’ link on the GenomicusPlant front page). More fragmented ancestral blocks or missing ancestral genes are often due to longer divergence time, to the quality of sequence assemblies and to the gene annotations of the extant genome species used to infer the ancestor. Divergence times displayed in GenomicusPlants were extract from the Timetree database (Hedges et al. 2006) when available or from the Angiosperm Phylogeny Website (Stevens 2001 onwards).

## Web Interface, Usage and Graphical Outputs

### Homepage

The home page of GenomicusPlants is divided into three parts. The left part allows an access to links about the server (the help menu that links a page with tutorial videos, Examples menu, Statistics and site history). The top of the right page is dedicated to the search panel. This search panel allows querying for a gene of interest by its name or by its ID (Ensembl Gene IDs or plant-specific gene IDs). The database can also be queried by a description word, and a selection of specific species. Once the user activates the ‘Go’ button, GenomicusPlants will load the default PhyloView, described in the next section. The home page allows a direct access to two pairwise genome comparison views, MatrixView and KaryoView.

### Pairwise genome comparison: MatrixView and KaryoView

**Fig. 2:**
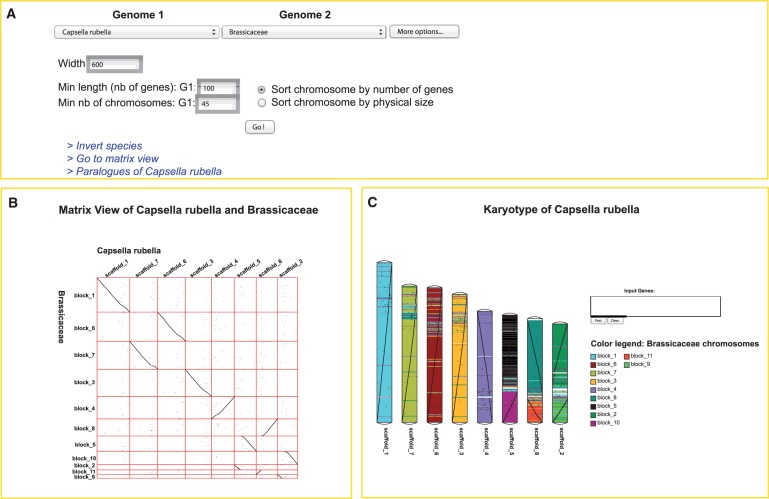
The two pairwise genome comparison modules, MatrixView and KaryoView. (A) The menu to select the two genomes (genome1 and genome2) is the same in the two modules, while three links allow users to switch between the two different views, to invert the two selected species and to jump to the dotplot of paralogs of genome1. Parameters such as the size and number of chromosomes to show can be adjusted here. (B) MatrixView is a dotplot of genome1 against genome2. Here, we represent the genome of *Capsella rubella* according to its ancestor Brassicaceae. (C) KaryoView allows users to visualize a genome (extant or ancestral) according to the colors of the syntenic chromosomes of a different genome (extant or ancestral). It can be seen that no important change in the structure of the *C. rubella* genome has occurred since its Brassicaceae ancestor. Two colors are used for scaffolds 5, 8 and 2 because their ancestral counterparts are each split over two reconstructed ancestral blocks.

The two different modules of pairwise genome comparison available in GenomicusPlants interactively display chromosome-scale synteny either between two extant genomes, between two ancestral genomes or between an ancestral and an extant genome. Fig. 2 describes the main top menu available in MatrixView or Karyoview to select the two genomes to compare. In this example, we selected the *Capsella rubella* genome against the reconstructed ancestor of all the Brassicaceae genomes present in the database.

The MatrixView is a classical dotplot module showing the relative order of genes between two plant genomes. It is a two-dimensional interactive image designed on the fly by requesting all orthologous genes between the two species, which are deduced from the computed gene trees stored in the database. Different actions can be performed on the matrix: (i) a user can hide or show one or more specific lines or columns of interest by a right mouse click on the name of the chromosome; (ii) each cell of the matrix can be zoomed-in by a simple mouse click; or (iii) once in the maximum zoomable box, the user can switch to the Phyloview module by selecting a gene.

The KaryoView module displays the karyotype of one species according to the chromosome color of the orthologous genes from another species. As for MatrixView, selecting or removing specific chromosomes is possible using a contextual menu available by a right mouse click. Note that while colors make it possible to show at once the chromosome-scale regions that share an evolutionary relationship between the two selected genomes, individual genes are also displayed as black dots along the chromosome axes, forming diagonals when their orders are conserved. Users may highlight the position of specific genes on the karyotype by entering their ID.

### Multiple genome comparison: PhyloView and AlignView

**Fig. 3:**
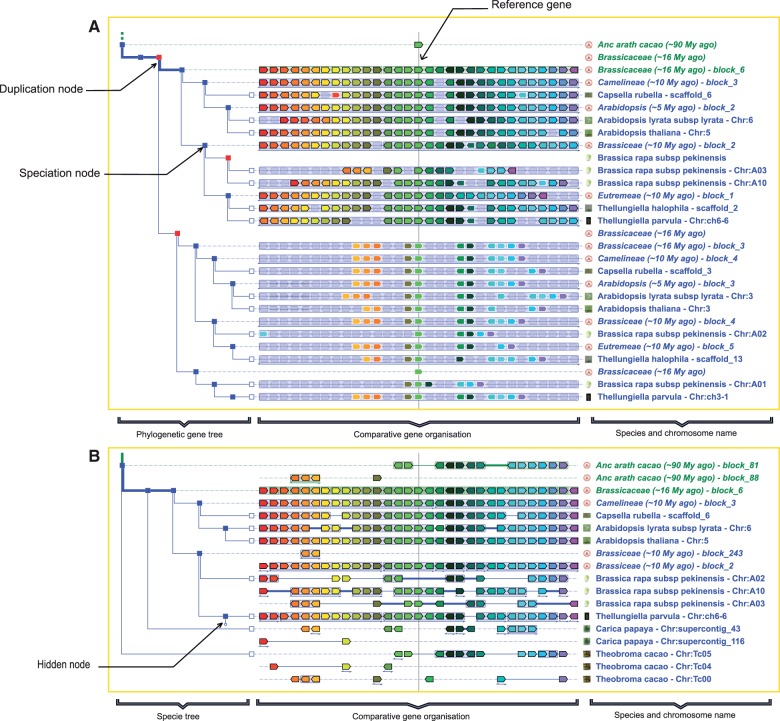
The two multiple genome comparison modules, PhyloView and AlignView. (A) The Phyloview of the ancestral gene TreeBeST009539.N.a on reconstructed block_6 of Brassicaceae. The duplication node (in red) represents the whole-genome duplication ‘alpha’ that occurred in the Brassicaceae clade 20–30 million years ago. By following the branches leading to the paralog copy on Brassicaceae block_3, one immediately sees that this locus contains many other paralogs of genes that are neighbors of TreeBeST009539.N.a (colored genes with white outlines). This strongly suggests that the two regions on Brassicaceae block_6 and block_3 originate from a single event that duplicated all the genes in the ancestral region. This situation is a typical signature of whole-genome duplication. (B) The AlignView of the gene TreeBeST009539.N.a on reconstructed block_6 of Brassicaceae. The hexapoliploidization event that occurred in *Brassica rapa* is clearly visible because the ancestral block_6 in the ancestral Brassicaceae genome now occurs three times in the extant *B. rapa* genome.

Once the user enters a reference gene in the home page, the PhyloView display (Fig. 3) shows the order of genes in its neighborhood, and the order of their orthologs and paralogs in all species where the reference gene exists (limited to 200 orthologs or paralogs by default). The lines are organized according to the phylogenetic tree displayed on the left part. Blue nodes represent speciation events and the red nodes represent duplication events.

The AlignView page shows an alignment between genes contained within the genomic region of the reference gene and all their respective orthologs in other species where they exist. Lines are organized according to the species tree and, in contrast to PhyloView, a genome without an ortholog of the reference gene can be displayed as long as it possesses orthologs of its neighboring genes. In short, AlignView displays the orthologous genomic environment of the reference gene in all the genomes that possess at least two collinear orthologous genes or more with the region of reference. Multiple lines are displayed for a genome if the reference region is distributed on several chromosomes (or several scaffolds when a chromosome assembly is not available). AlignView is very useful to infer gene losses or gene gains during evolution, or to confirm the presence of an evolutionary breakpoint.

In both PhyloView and AlignView, the reference gene and its homologs are displayed on the vertical line in the middle of the page, always in green. The reference genome is always located at the leaf of the branches shown in bold, with the name of the species written in green. Each node can be collapsed or expanded; branches can be hidden or shown by a simple click. Finally, a contextual selection box can be opened on the left of the page to select the list of genomes (from extant or ancestral species) to be displayed.

## Menus and Tips

The top menu of PhyloView and AlignView provides information on the reference gene or on the selected gene in the graphical display. The user can then change the reference gene or switch to a different reference genome. The menu allows users to switch between the two types of multispecies comparison views, focusing only on paralogs of the reference gene, hiding all ancestral species or all outgroups of a specific species. Data can be exported in text format or as an SVG (Scalable Vector Graphics) image that can be edited in Inkscape for editing.

## Database and Website Implementation

GenomicusPlant is developed using the client/server model. On the server side, we use MariaDB (version 5.5) as the database server to store all the GenomicusPlant’s data, Apache2 (version 2.2) as the web server, and Perl (version 5.8) as the language for implementing CGI-scripts. Apache2 runs with the mod_perl module to process perl scripts to render HTML content for displaying on the client side. Graphic contents are generated with inline SVG drawings in XHTML. This allows exportation in vectorial format for future edition. On the client side, the interactions between users and the web interface are supported by Asynchronous Javascript and XML calls (AJAX). The interface is optimized for Firefox and Chrome Navigators. It can also run on Safari and Internet Explorer. Genomicus source codes and MariaDB schema are available on request.

## Future Plans

At the present time, GenomicusPlants focuses on angiosperms. We plan to build automatic updates of the ancestral genome reconstructions based on species stored in the EnsemblGenome-Plant database, as is done for the vertebrate Genomicus server. Other clades would then be available in GenomicusPlants, such as gymnosperms and bryophytes. Additional information would then easily be added such as sequence similarity between orthologs and paralogs and dN/dS information, both shown as a color gradient as in the Genomicus server dedicated to Vertebrates.

## Funding

This study was suppported by the Centre National de la Recherche Scientifique (CNRS); Agence Nationale de la Recherche [Ancestrome Project ANR-10-BINF-01-03, ANR Blanc-PAGE ANR-2011-BSV6-00801]. Funding for open access charges is provide by the CNRS.

## Abbreviations

AGORA: Algorithm for Gene Order Reconstruction in Ancestors
AJAX: Asynchronous JavaScript and XML
API: Application Programming Interface
CGI: Common Gateway Interface
SVG: Scalable Vector Graphics
XHTML: Extensible HyperText Markup Language
